# Transcriptome Analysis Reveals Key Genes Involved in Fatty Acid and Triacylglycerol Accumulation in Developing Sunflower Seeds

**DOI:** 10.3390/genes16040393

**Published:** 2025-03-29

**Authors:** Wanqiu Meng, Linglu Zeng, Xiuli Yang, Dawei Chen, Li Sun

**Affiliations:** College of Life Science, Shihezi University, Shihezi 832000, China; 18899537998@163.com (W.M.); lingluzeng@163.com (L.Z.); 15899050870@163.com (X.Y.); 20211006030@stu.shzu.edu.cn (D.C.)

**Keywords:** sunflower (*Helianthus annuus* L.), developing seeds, transcriptome, lipid biosynthesis, unsaturated fatty acids

## Abstract

Background/Objectives: Sunflower (*Helianthus annuus* L.) is one of the four major global oilseed crops. Understanding the molecular mechanisms regulating fatty acid synthesis and triacylglycerol (TAG) accumulation is crucial for improving oil yield and quality. In this study, the oilseed sunflower cultivar ‘T302’, which was wild-cultivated in the northwestern region of China, was analyzed for fatty acid content by targeted lipidomic analysis. RNA sequencing (RNA-seq) was performed on 15 cDNA libraries from sunflower embryos at five developmental stages (10, 17, 24, 31, and 38 days after flowering) to investigate gene expression patterns during oil accumulation. Differentially expressed genes (DEGs) related to fatty acid and triacylglycerol accumulation in developing sunflower seeds were identified. WGCNA was used to gain deeper insights into the mechanisms underlying lipid metabolism. Results: The oil composition of ‘T302’ consisted of 86.61% unsaturated fatty acids (UFA), mainly linoleic acid (48.47%) and oleic acid (37.25%). Saturated fatty acids (SFAs) accounted for 13.39%, with palmitic acid (7.46%) and stearic acid (5.04%) being the most abundant. A total of 81,676 unigenes were generated from RNA-seq data, and 91 DEGs associated with lipid metabolism were identified, including key enzymes such as *FAD2-1*, *SAD*, *FATA*, *LACS*, *PDAT2*, and *DGAT2*. In addition, we identified several novel candidate transcription factor genes, including *WRI1*, *LEC1*, *FUS3*, and *ABI3*, which were found to regulate TAG synthesis during seed maturation and are worthy of further investigation. This study provides valuable insights into the molecular mechanisms of seed oil biosynthesis in oilseed sunflower. The identified key genes and transcription factors provide potential targets for molecular breeding strategies to increase oil content and modify fatty acid compositions in sunflower and other oilseed crops.

## 1. Introduction

Sunflower (*Helianthus annuus* L.) is one of the four major oil crops worldwide and plays a significant role in global agriculture. Sunflower seeds contain abundant vegetable oils and proteins that are essential for human nutrition and are widely used in the food sector [1,2]. The fatty acid (FA) composition of traditional sunflower seed oil is characterized by a high concentration of unsaturated fatty acids (UFAs) (approximately 85%), predominantly oleic acid (OA, C18:1; 14–43%) and linoleic acid (LA, C18:2; 44–75%). The remaining 15% consists of saturated FAs, primarily palmitic acid (PA, C16:0) and stearic acid (SA, C18:0) [3]. In recent years, sunflower breeding has resulted in the development of high-quality oils with different OA levels, including medium OA (43.1–71.8%) and high OA (75–90.7%) sunflower hybrids, which contain more OA than traditional sunflower varieties [4]. Edible oils rich in UFAs are healthy for humans, which can lower blood pressure and lipid levels, thus providing protective benefits for cardiovascular and cerebral vessels [5,6]. Therefore, increasing seed oil content and optimizing FA profiles are key objectives in sunflower breeding programs to improve crop quality.

Lipid biosynthesis in plants consists of four interrelated processes: the de novo synthesis of FAs, acyl elongation and modification, triacylglycerol (TAG) formation, and oil droplet production [7,8]. De novo FA biosynthesis takes place in plastids through a series of reactions, including condensation, reduction, and dehydration processes, facilitated by fatty acid synthases (FAS). As a result, free FAs are generated, including PA, SA, and OA [9]. A number of enzymes participate in this process, such as acetyl-CoA carboxylase (ACC), 3-ketoacyl-ACP synthase (KAS), β-ketoacyl-ACP reductase (KAR), β-hydroxyacyl-ACP dehydratase (HAD), and enoyl-ACP reductase (ENR). Additionally, acyl-ACP thioesterases (FATA/B) and stearoyl-ACP desaturase (SAD) play crucial roles in FA modification and release [10,11]. After synthesis, long-chain free FAs (C16→18) are exported from the plastid to the endoplasmic reticulum (ER), where they undergo further elongation, acyl modification, and lipid assembly [12]. At the outer envelope of the plastid, long-chain acyl-CoA synthetases (LACS) convert free FAs into fatty acyl-CoA, which is the essential substrate for glycerolipid assembly [7].

In plants, TAGs are produced in the ER through two main pathways: an acyl-CoA-dependent pathway and an acyl-CoA-independent pathway [13]. The acyl-CoA-dependent pathway, or Kennedy pathway, utilizes acyl-CoA as a substrate to assemble TAG. Several key enzymes are involved in this process, including glycerol-3-phosphate acyltransferase (GPAT), lysophosphatidic acid acyltransferase (LPAAT), phosphatidic acid phosphatase (PAP), and diacylglycerol acyltransferase (DGAT), which work together to acylate the sn-3 position of diacylglycerol (DAG), leading to the formation of TAG [14,15]. In the acyl-CoA-independent pathway, phospholipid: diacylglycerol acyltransferase (PDAT) catalyzes the transfer of an acyl group from phosphatidylcholine (PC) to DAG, leading to the formation of TAG [16]. The relative contributions of DGAT and PDAT to TAG biosynthesis remain unclear. During the final stages of oil synthesis, key oil body-associated proteins, including oleosin, caleosin, and steroleosin, bind to TAG molecules to form oil bodies [17].

In the lipid biosynthetic pathway of plants, the content and catalytic activity of fatty acid desaturases (FADs) are critical determinants of the composition and ratio of UFAs [18]. FADs can be classified into two main groups based on their localization and associated cofactors: soluble desaturases and membrane-bound desaturases. Soluble desaturases, such as SAD, are the only known enzymes that convert stearic acid (18:0-ACP) to oleic acid (18:1-ACP), a key step in UFA synthesis [19]. Membrane-bound desaturases, attached to plastid or ER membranes, consist of seven main types: FAD2, FAD3, FAD4, FAD5, FAD6, FAD7, and FAD8 [12]. The ω-6 desaturases, FAD2 and FAD6, add double bonds at the ω-6 (Δ-12) position, converting OA into LA. On the other hand, ω-3 desaturases, including FAD3, FAD7, and FAD8, introduce double bonds at the ω-3 (Δ-15) position, converting LA into linolenic acid (ALA, 18:3) [15].

In addition, several transcription factors (TFs), including WRI1 (WRINKLED1), LEC1 (Leafy cotyledon 1), LEC2, ABI3 (abscisic acid insensitive 3), and FUS3 (FUSCA3), play key roles in regulating embryo development [20]. WRI1, a TF of the APETALA2/ethylene-responsive element binding factor (AP2/ERF) [21], plays a crucial role in regulating FA synthesis. In *Arabidopsis thaliana*, AtWRI1 controls oil accumulation by regulating the carbon flux from sugars to oils [22,23]. LEC1, a member of the NF-YB family, contributes to FA biosynthesis through synergistic interactions with TFs such as ABI3 and WRI1 [24]. FUS3 activates the expression of storage proteins, including oleosin, and lipid synthesis genes, by physically cooperatively with LEC1 and LEC2, which belong to the B3 family, in a cooperative manner [25]. Alterations such as mutations, overexpression, or heterologous expression of these TFs greatly influence seed formation, FA synthesis, and oil content [26,27,28].

In recent years, significant progress has been made in the improvement of the quality of sunflower oil. An integrated quantitative genetics approach has established a gene network for sunflower oil metabolism that includes 429 genes linked to 125 reactions in 12 pathways. Notably, 46 oil-related genes were identified within 32 genomic regions, facilitating the discovery of candidate genes for enhancing sunflower oil quality [29]. Seven quantitative trait loci (QTLs) related to OA, total oil, and LA content were identified, explaining over 10% of the phenotypic variation in sunflower traits [30]. In addition, genetic variation related to FA content has been identified in Russian sunflower collections through genome-wide association studies (GWAS) combined with high-throughput lipidome phenotyping [31]. Transcriptome studies on UFA biosynthesis suggest that OA synthesis plays a dominant role in UFA biosynthetic pathways in seed embryos [32].

Despite advancements in sunflower oil metabolism, several gaps remain in current research. Seed oil yield and FA profile are complex traits influenced by genetic, physiological, biochemical, and environmental factors. However, the precise molecular mechanisms underlying TAG accumulation and unsaturated FA biosynthesis in oilseed sunflower are still poorly understood. Additionally, the transcriptional regulatory mechanisms of these processes remain unclear, limiting the potential for optimizing sunflower oil production and quality through genetic engineering. Therefore, the study of the total gene expression of RNA transcripts in sunflower seeds is crucial for advancing our understanding of the molecular mechanisms involved in FA biosynthesis. In this study, seeds of sunflower cultivar ‘T302’ within five developmental stages were used for transcriptome analysis by Illumina sequencing technology. The key genes and transcription factors involved in FA biosynthesis were identified. The results will increase our knowledge of the molecular mechanisms of FA synthesis and oil accumulation in sunflower seeds and provide a basis for future advances in sunflower breeding and metabolic engineering.

## 2. Materials and Methods

### 2.1. Plant Materials

The hybrid oilseed sunflower cultivar ‘T302’, which is widely cultivated in the northwestern region of China, was used as the plant material. This culture is curated by the College of Life Sciences, Shihezi University, Xinjiang, China. The seeds were grown in May 2020 in the experimental field of Shihezi (44°18′ N, 86°00′ E), on soils composed mainly of gray desert soil and irrigated soil, with summer daylight lasting 12–14 h and regular irrigation. Nylon mesh bags were used to cover the flower heads before flowering to ensure that the seeds obtained were self-pollinated. Embryo samples were collected from 10 days after anthesis (DAF), three flower heads were sampled every 7 days until 38 DAF, and seeds were collected from the outer three whorls of each flower head. At each developmental stage, the seed coats were carefully removed from the embryos, with a total of 20 embryos collected per plant, immediately frozen in liquid nitrogen, and stored at −80 °C.

### 2.2. Lipid Extraction and Targeted Lipidomic Analysis

Forty fatty acid methyl ester (FAME) standards were prepared in ten concentration gradients (0.5–1000 mg/L), with each gradient representing the total concentration of all components. In the 40 FAME standards, the concentration of each component was either 2% or 4% of the total, with 30 components at 2% and 10 components at 4%. For sample analysis, 500 μL of the FAME standard was combined with 25 μL of methyl nonadecanoate (500 ppm) as an internal standard. The mixture was injected (1.0 μL) into a Gas Chromatography–Mass Spectrometry (GC-MS) system with a 10:1 split ratio.

Mature ‘T302’ sunflower seeds (50 mg) were placed in 2.0 mL glass centrifuge tubes, and 1.0 mL chloroform-methanol (2:1, v/v) was added and sonicated for 30 min. The supernatant was collected and methylated by adding 2.0 mL of a 1% sulfuric acid–methanol solution, then incubated at 80 °C for 30 min. After cooling, 1.0 mL of hexane was added to extract the lipids, and the organic phase was washed with 5.0 mL of deionized water. A 500 μL portion of the supernatant was mixed with 25 μL of methyl salicylate (internal standard) and transferred to a vial. Finally, 1.0 μL of the sample was injected into the GC-MS system with a 10:1 split ratio.

GC-MS analysis was performed by Shanghai Zhongke Xinlife Biotechnology Co., Ltd. (Shanghai, China) using an Agilent 7890/5975C GC-MS system. The samples were analyzed using an Agilent DB-WAX capillary column (30 m × 0.25 mm ID × 0.25 μm) in a gas chromatography system. The initial temperature was set at 50 °C and held for 3 min, then ramped to 220 C at a rate of 10 °C/min and held for 5 min. Helium was used as the carrier gas at a flow rate of 1.0 mL/min. A quality control (QC) sample was analyzed periodically to evaluate system stability and reproducibility. The injector temperature was set at 280 °C and electron ionization (EI) was performed at 70 eV. Data were acquired using MSD ChemStation software (version E.02.00 Keysight Technologies) to measure peak areas and retention times. A standard curve was constructed for the quantification of long chain fatty acids (FAs), and three biological replicates were analyzed to determine the FA profile of sunflower cultivar ‘T302’.

### 2.3. RNA Extraction, cDNA Library Construction, and Transcriptome Sequencing

RNA extraction, cDNA library construction, and sequencing were carried out by Shanghai Meiji Biomedical Technology Co., Ltd (No. 3, Lane 3399, Kangxin Road, Pudong New Area, Shanghai, China). Total RNA was isolated from sunflower embryos with TRIzol reagent (Invitrogen, Carlsbad, CA, USA) and then treated with DNase I to eliminate genomic DNA contamination. Five developmental stages of embryos were selected for RNA sequencing (RNA-seq): 10, 17, 24, 31, and 38 DAF, designated S1 (stage 1), S2 (stage 2), S3 (stage 3), S4 (stage 4), and S5 (stage 5), respectively. RNA samples were sequenced on the NovaSeq 6000 Sequencing System (Illumina), with three independent biological replicates.

Raw data were cleaned by removing reads containing adapters, poly-N sequences, and low-quality reads. Analysis of the cleaned data was performed using HISAT2 (v2.0.5) [33] and StringTie [34]. Sequences were aligned to the sunflower genome [29] (https://www.ncbi.nlm.nih.gov/datasets/genome/GCF_002127325.2/, accessed on 12 November 2022) using the HISAT2 transcript program. The raw sequencing data have been deposited in the Sequence Read Archive (SRA) under the NCBI BioProject, with accession number PRJNA1220589.

### 2.4. Sequence Alignment and Functional Annotation

The clean reads, obtained after quality control, were aligned to the *H. annuus* reference genome (https://www.sunflowergenome.org/annotations-data/ accessed on 22 March 2025). The mapped data were used for subsequent transcript assembly, expression quantification, and alignment against six databases: NR (NCBI non-redundant protein sequences), Pfam (protein family), Swiss-Prot (annotated and reviewed protein sequence database), COG (Clusters of Orthologous Groups of Proteins), GO (Gene Ontology), and KEGG (Kyoto Encyclopedia of Genes and Genomes). Unigenes identified by BLASTx (version 2.9.0) (e-value < 1 × 10^−5^) were annotated using BLAST2GO to describe biological processes (BP), cellular components (CC), and molecular functions (MF).

### 2.5. Differentially Expressed Genes (DEGs) Analysis

Clean reads were aligned to assembled unigenes using Bowtie2 (version 2.4.1) [35], and gene expression was quantified in FPKM (fragments per million reads per mapped kilobase). DEGs were identified using DESeq2 (version 1.24.0) [36] with an adjusted *p*-value < 0.05 and |log2FC| ≥ 1 as thresholds. GO and KEGG enrichment analyses were performed on DEGs, with FDR ≤ 0.001 considered significant. TFs were predicted by PlantTFDB 5.0 (http://planttfdb.gao-lab.org/ accessed on 22 March 2025).

### 2.6. Co-Expression Network Construction

Gene co-expression networks were constructed using WGCNA with hybrid network types (https://horvath.genetics.ucla.edu/html/CoexpressionNetwork/Rpackages/WGCNA/ accessed on 22 March 2025). Module eigengene (ME), representing the first principal component of each module, was used to evaluate the correlation with oil content by calculating the values for each module.

### 2.7. Quantitative Reverse Transcriptase Polymerase Chain Reaction (RT-qPCR) Analysis

RT-qPCR was used to validate gene expression changes at three developmental stages: 10, 24, and 38 DAF. Five micrograms of total RNA was synthesized into cDNA using PrimeScript RT reagents (Kusatsu City, Japan, Takara Bio Inc.). Gene expression analysis was performed using SYBR Green I (CWBIO) on a LightCycler 480 system with three biological replicates. The sunflower 18S rRNA gene (AF1057577) was used as an internal control [37]. The specific primer sequences used are listed in Table S1. Relative gene expression levels were calculated using the 2^−ΔΔCT^ method [38].

### 2.8. Statistical Analysis

Statistical analysis was performed using SPSS 25.0 software. Data are presented as mean ± standard deviation (SD). One-way ANOVA followed by Tukey’s post hoc test was used to evaluate differences between groups, with a significance threshold of α = 0.05. Groups with different letters (e.g., ‘a’ and ‘b’) indicate statistically significant differences (*p* < 0.05). Before analysis, normality was assessed using the Shapiro–Wilk test and homogeneity of variance was confirmed using Levene’s test.

## 3. Results

### 3.1. Fatty Acid Composition in Mature Sunflower Seeds

The oil composition of sunflower cultivar ‘T302’ showed a high percentage of UFAs, accounting for 86.61% of the total oil (Table 1). UFAs were further divided into monounsaturated fatty acids (MUFAs) and polyunsaturated fatty acids (PUFAs). LA was the predominant PUFA (48.47%), and OA was the predominant MUFA (37.25%). Small amounts of several UFAs were also present. Saturated fatty acids (SFAs) were present at 13.39%, with PA (7.46%) and SA (5.04%) being the most abundant SFAs. In addition, trace amounts of nine other SFAs were detected.

### 3.2. Illumina Sequencing and De Novo Assembly

A total of 105.15 GB of raw paired-end data were obtained from the transcriptome sequencing. Quality control analysis revealed a low error rate of 0.02%, with over 97% of reads achieving Q20 and over 92% achieving Q30, along with an average GC content of 47.36%. After filtering out low-quality reads, adapter contamination, and sequences with a high number of unknown bases (N), a total of 81,676 unique transcripts were obtained (Table S2), with clean reads accounting for over 81.65% of the total. Among these, 27,796 unigenes were longer than 1800 base pairs (bp) (Figure S1).

### 3.3. Gene Functional Annotation

The coding sequences (CDS) and corresponding amino acid sequences of 81,676 non-redundant unigenes were annotated. BLAST alignments (E-value ≤ 10^−5^) were performed against six public databases: NR, GO, KEGG, COG, Pfam, and Swiss-Prot. The results showed that 12,361 unigenes (20.50%) were annotated in the GO database; 16,022 (26.58%) in KEGG; 40,205 (66.69%) in COG; 43,843 (72.72%) in NR; 31,691 (52.57%) in Swiss-Prot; and 36,749 (60.96%) in Pfam (Figure S2).

To assess the functional roles of the unigenes, we performed a classification using the COG database. A total of 63,478 unigenes were classified into 23 functional categories. Excluding the category “[S] Function unknown”, the largest group was “[L] Replication, recombination, and repair” (10,081 unigenes, 15.88%), followed by “[O] Post-translational modification, protein turnover, chaperones” (2951 unigenes, 4.65%). Notably, 795 unigenes (1.25%) were categorized under “[I] Lipid transport and metabolism”, suggesting that these candidate unigenes may be involved in lipid metabolism in sunflower seeds (Figure S3).

To investigate their functions, a total of 34,781 unigenes were annotated in the GO database. They were grouped into three main categories: molecular functions, biological processes, and cellular components (Figure S4), and further classified into 11, 15, and 14 subcategories, respectively. In the molecular functions category (13,887 unigenes), the most prominent subcategory was “binding” (5749 unigenes, 41.40%), followed by “catalytic activity” (6352 unigenes, 45.74%). In the biological process category (17,897 unigenes), “metabolic process” (6231 unigenes, 34.82%) and “cellular process” (5740 unigenes, 32.07%) were the dominant subcategories. Within the cellular component category (16,884 unigenes), the most abundant subcategory was “cell part” (5288 unigenes, 31.32%). These results suggest that metabolic and enzymatic activities play an important role during sunflower seed development.

**Figure 1:**
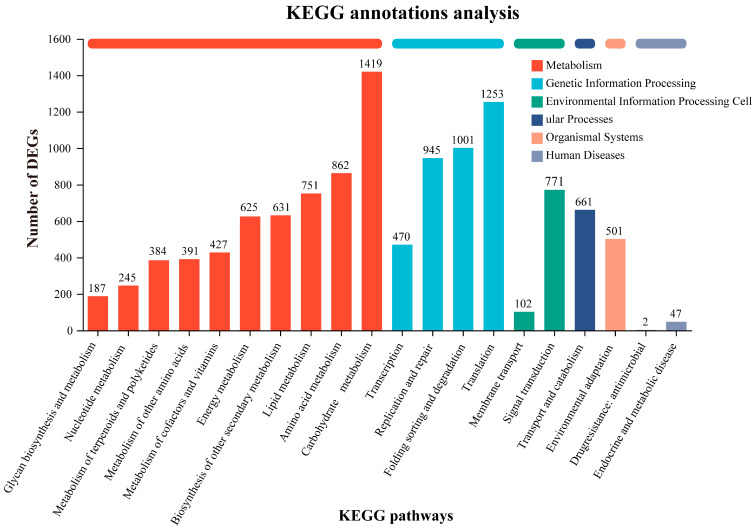
KEGG pathway enrichment analysis of the unigenes. The *X*-axis represents the names of the KEGG metabolic pathways, while the *Y*-axis denotes the count of genes or transcripts annotated to each respective pathway.

To further explore the potential pathways of these unigenes, a KEGG pathway analysis was performed (Figure 1). A total of 15,550 unigenes were assigned to 20 subcategories in six KEGG categories, corresponding to 136 KEGG pathways. “Metabolism” was the largest category with 5922 unigenes, followed by “Genetic Information Processing” (3669 unigenes). The most prominent subcategories were “carbohydrate metabolism” (1419 unigenes) and “translation” (1253 unigenes). Notably, 751 unigenes were annotated under “lipid metabolism”, which was similar to the COG results of 795 unigenes, providing valuable insights into fatty acid and oil accumulation during sunflower seed development. The 20 pathways were grouped into six major categories, with three key pathways standing out: “replication and repair” (2859 unigenes), “carbohydrate metabolism” (2017 unigenes), and “translation” (1343 unigenes). Of the total 15,550 unigenes, approximately 897 were involved in 14 key pathways of lipid metabolism. Among these, “glycerophospholipid metabolism” contained the largest number of unigenes, followed by “α-linolenic acid metabolism”, “FA degradation”, and “FA biosynthesis”. Other lipid metabolism-related pathways included “LA metabolism”; “FA elongation”; “UFA biosynthesis”; and “keratin, chondroitin, and wax biosynthesis”. These findings highlight unigenes that are crucial for identifying key genes involved in FA and TAG biosynthesis in sunflower seeds, providing a basis for further investigation of lipid accumulation processes.

### 3.4. Analysis of Differentially Expressed Genes in Developing Sunflower Seeds

**Figure 2:**
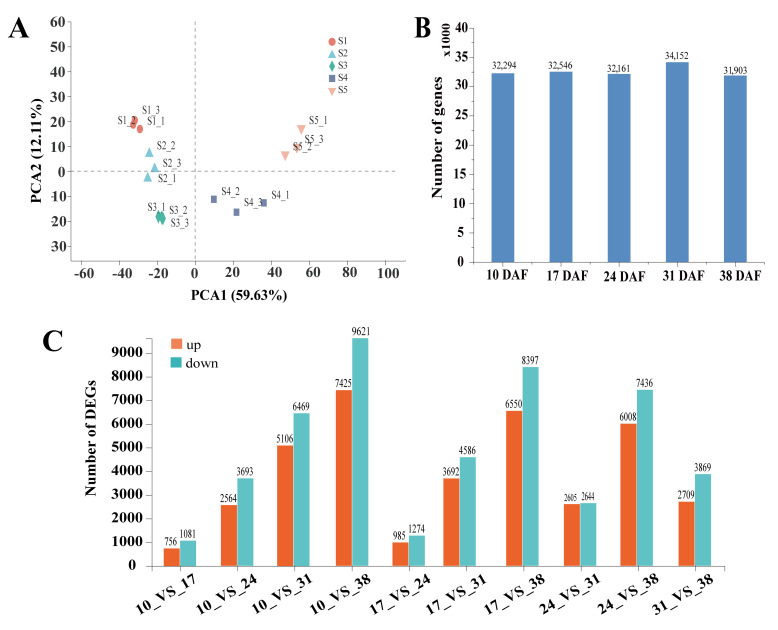
PCA analysis of sunflower transcriptome samples and cluster analysis of DEGs. (**A**) Principal component analysis (PCA) of all 15 samples was performed based on RNA-seq FPKM. (**B**) The number of genes in sunflower seeds at different developmental stages: 10, 17, 24, 31, and 38 days after flowering (DAF). (**C**) Pairwise comparisons of the number of DEGs between samples at each time point during sunflower seed development were performed. A *p*-value < 0.05 and a fold change ≥ 1 were used as criteria for DEG identification.

A total of 81,676 unigenes were identified from the transcriptome data. Principal component analysis (PCA) of the 15 samples based on RNA-seq FPKM values showed that two principal components explained 71.74% of the total variance (59.63% for PC1 and 12.11% for PC2). The samples were clearly clustered into five groups, each corresponding to a specific developmental stage, demonstrating consistency among the three biological replicates collected for each stage (Figure 2A). The number of genes expressed at each stage was determined based on gene expression levels (Figure 2B). A total of 23,396 DEGs were identified by pairwise comparisons of expression levels between samples at each time point (Table S3). Using the samples at 10, 17, and 24 DAF as controls, the number of DEGs identified at different time points can be observed in Figure 2C. The highest number of DEGs was observed at 10 DAF vs. 38 DAF, with 7425 up- and 9621 down-regulated unigenes. The second largest difference occurred at 17 DAF vs. 38 DAF with 6550 up- and 8379 down-regulated unigenes (*p*-value < 0.05).

To explore the DEGs related to FA synthesis and triacylglycerol accumulation during sunflower seed development, KEGG and GO enrichment analyses were performed on the 23,396 DEGs, and 522 DEGs related to lipid metabolism were identified. These genes participated in various pathways, such as glycerolipid metabolism, glycerophospholipid metabolism, sphingolipid metabolism, FA biosynthesis, elongation, degradation, and UFA biosynthesis, including α-linolenic acid, arachidonic acid, and LA metabolism (Table S4; Figure S5).

### 3.5. Weighted Gene Co-Expression Network Analysis

**Figure 3:**
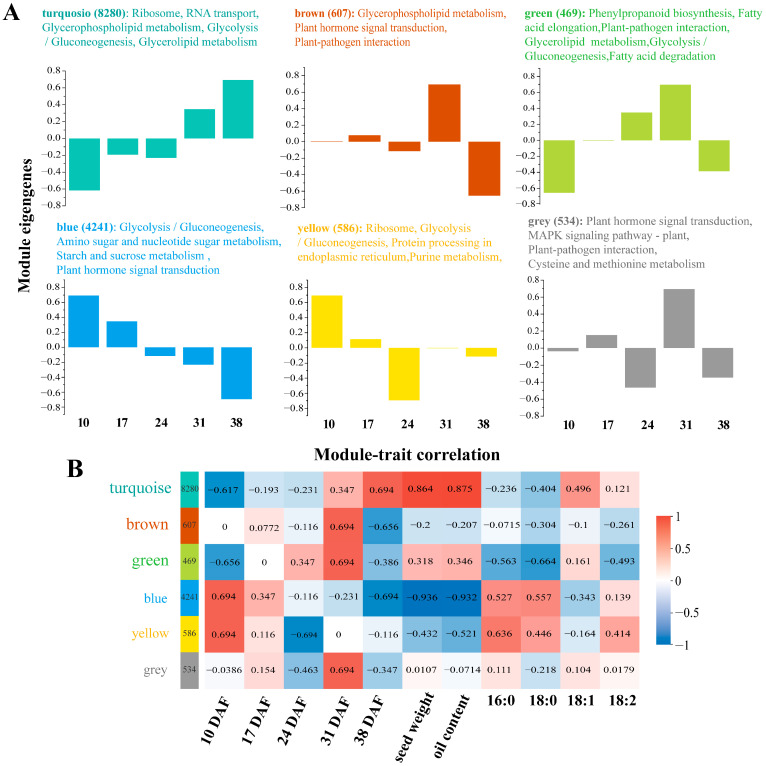
WGCNA analysis of RNA-seq and physiological trait data. (**A**) WGCNA was calculated from 15 samples, and the 23,396 DEGs were partitioned into six modules. Columns represent the module eigengenes of the means. For each module, the number of DEGs and the number of KEGG pathways were listed. (**B**) The expression profiles of the modules were related to the date of sampling (DAF) and the physiological traits in Figure 1. The data in the boxes represent the number of differentially expressed genes.

To gain deeper insights into the mechanisms underlying lipid metabolism in sunflower, we analyzed the trends in DEGs. The transcriptomic changes were evaluated using weighted gene co-expression network analysis (WGCNA), which revealed six distinct gene modules (Figure 3A). For each module, the number of DEGs and the corresponding KEGG pathways were characterized. KEGG enrichment analysis identified 282 DEGs associated with lipid metabolism by WGCNA (Table S5).

The turquoise-colored module, the largest identified, contained 8280 DEGs with peak expression at 38 DAF. This module included genes involved in ribosome function, RNA transport, glycerophospholipid metabolism, glycolysis, and glycerolipid metabolism. The second largest module, colored blue, contained 4241 DEGs with peak expression at 10 DAF. Genes in this module were associated with glycolysis, amino and nucleotide sugar metabolism, starch and sucrose metabolism, and phytohormone signaling. The brown-colored module contained 607 DEGs that were up-regulated at 31 DAF and down-regulated at 38 DAF. These genes are related to glycerophospholipid metabolism, phytohormone signaling, and plant–pathogen interactions. The yellow-colored module, consisting of 586 DEGs, showed peak expression at 10 DAF. This module included genes involved in ribosome function, glycolysis, protein processing in the ER, and purine metabolism. The gray-colored module contained 534 DEGs with peak expression at 31 DAF. These genes are involved in phytohormone signaling, the MAPK pathway, plant–pathogen interactions, and cysteine and methionine metabolism. The smallest module, the green-colored module, contained 469 DEGs that were up-regulated from 17 to 31 DAF. These genes are associated with phenylpropanoid biosynthesis, FA elongation, plant–pathogen interactions, glycerolipid metabolism, glycolysis, and FA degradation.

To explore the relationship between module gene expression patterns and physiological traits, WGCNA was performed (Figure 3B). The accumulation rates of PA and SA were strongly correlated with the blue- and yellow-colored modules, respectively, while OA content showed a significant association with the turquoise-colored module. This module was also closely related to seed weight and oil content, while LA content showed a predominant correlation with the yellow-colored module. Together with KEGG pathway and module–trait correlation analyses, these results suggest that the turquoise-, blue-, and yellow-colored modules play key roles in FA biosynthesis.

### 3.6. Identification of Genes Involved in Lipid Biosynthesis

**Figure 4:**
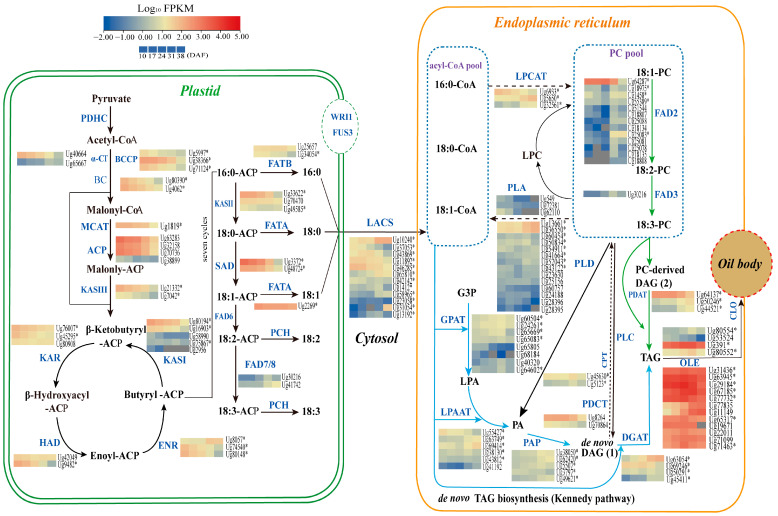
Fatty acid and TAG biosynthetic pathways in sunflower seeds. The expression levels of potential candidate genes (represented by Log10 FPKM) in sunflower seeds at various developmental stages (10, 17, 24, 31, and 38 DAF) are highlighted through color coding, ranging from blue (low expression) to red (high expression). An asterisk (*) denotes the differentially expressed genes (DEGs) associated with FA synthesis, as identified through WGCNA analysis. Key genes involved in lipid metabolism include *ACCase* (acetyl-CoA carboxylase), *BC* (biotin carboxylase), *BCCP* (biotin carboxyl carrier protein), *α-CT* (carboxyl transferase subunit α), *MACT* (malonyl-CoA-acyl carrier protein transacylase), *ACP* (acid phosphatase), *KAS III* (3-oxoacyl-ACP synthase III), *KAR* (3-oxoacyl-ACP reductase), *HAD* (3-hydroxyacyl-ACP dehydratase), *ENR* (Enoyl-CoA reductase), *KAS II* (3-oxoacyl-ACP synthase II), *FATB* (fatty acyl-ACP thioesterase B), *FATA* (fatty acyl-ACP thioesterase A), *SAD* (stearoyl-ACP desaturase), *FAD* (fatty acid desaturase), *LACS* (long-chain acyl-CoA synthetase), *LPCAT* (lysophosphatidylcholine acyltransferase), *PLA2* (phospholipase A2), *GPAT* (glycerol-3-phosphate acyltransferase), *LPAAT* (1-acyl-sn-glycerol-3-phosphate acyltransferase), *PLD* (phospholipase D), *PAP* (phosphatide phosphatase), *CPT* (CDP-choline: diacylglycerol cholinephosphotransferase), *PDCT* (phosphatidylcholine: diacylglycerol cholinephosphotransferase), *PLC* (phospholipase C), *DGAT* (diacylglycerol acyltransferase), *PDAT* (phospholipid: diacylglycerol acyltransferase), *OLE* (oleosin), *CLO* (caleosin), *WRI1* (wrinkled 1), and *FUS3* (FUSCA3).

In this study, we identified 138 DEGs related to FA and TAG biosynthesis from a total of 522 lipid metabolism-related DEGs, as determined by GO and KEGG analysis. These genes are involved in processes such as FA initiation, elongation, desaturation, thioesterase activity, acyl-CoA activation, Kennedy pathway, TAG synthesis, phospholipase activity, TAG lipase activity, and oil body formation (Table S6). Subsequently, by integrating 282 lipid metabolism-related DEGs identified by WGCNA, we finally identified 91 critical DEGs related to FA and TAG biosynthesis and oil bodies (Table 2). A model of lipid biosynthesis in the sunflower embryo is shown in Figure 4.

#### 3.6.1. Genes Involved in Fatty Acid Biosynthesis in Plastids

**Figure 5:**
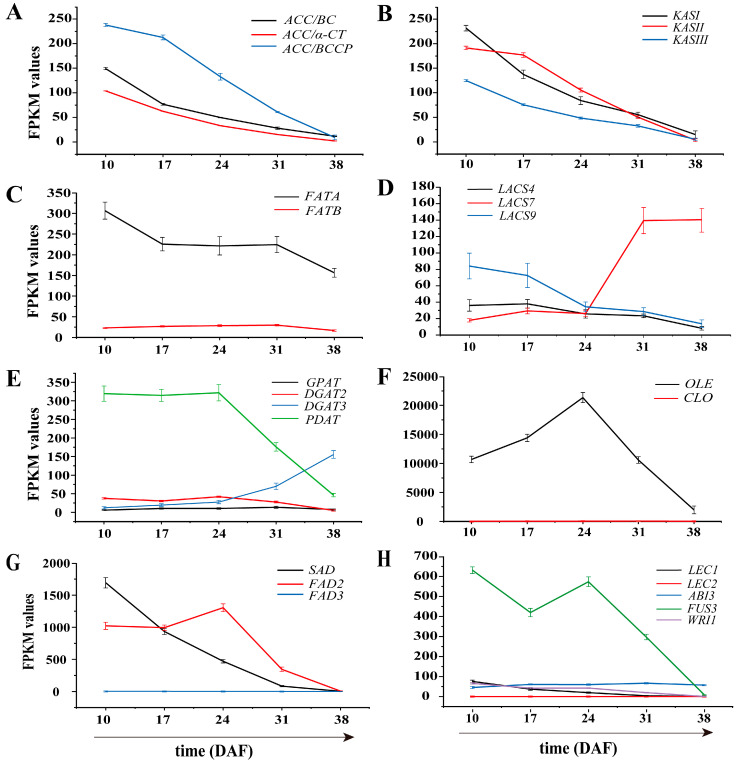
DEGs related to FA and TAG biosynthetic pathways identified in developing sunflower seeds. The Y-axis represents reads per kilobase per million mapped reads (FPKM) values and the X-axis represents five developmental stages of sunflower seeds: 10, 17, 24, 31, and 38 days after anthesis (DAF). The error bar line represents the standard deviation (SD) calculated from three biological replicates. (**A**) Acetyl-CoA carboxylase subunits (*BC*, *BCCP*, *α-CT*); (**B**) β-Ketoacyl-ACP synthase isoforms (*KASI*, *KASII*, *KASIII*); (**C**) Acyl-ACP thioesterases (*FATA*, *FATB*); (**D**) Long-chain acyl-CoA synthetases (*LACS4*, *LACS7*, *LACS9*); (**E**) Triacylglycerol assembly enzymes (*GPAT*, *DGAT2*, *DGAT3*, *PDAT*); (**F**) Oil body-associated proteins (*OLE*, *CLO*); (**G**) Fatty acid desaturases (*SAD*, *FAD2*, *FAD3*); (**H**) Seed development regulators (*LEC1*, *LEC2*, *ABI3*, *FUS3*, *WRI1*).

FA biosynthesis in plants begins in the plastids, where pyruvate is converted to acetyl-CoA, the primary substrate for FA synthesis. Acetyl-CoA carboxylase (ACC) catalyzes the carboxylation of acetyl-CoA to malonyl-CoA, the first rate-limiting step in the FA biosynthetic pathway. ACC is a multi-subunit enzyme consisting of biotin carboxyl carrier protein (BCCP), biotin carboxylase (BC), α-carboxyltransferase (α-CT), and β-carboxyltransferase (β-CT). Our analysis showed that *BC*, *BCCP*, and *α-CT* were predominantly highly expressed during the early to mid-stages of sunflower embryo development, with expression levels decreasing in the late stages of embryo development (Figure 4 and Figure 5A; Table 2).

Plastid FA synthesis involves three KAS enzymes, each with a different role. KASIII initiates the condensation reaction by combining acetyl-CoA and malonyl-ACP to form 4:0-ACP. KASI elongates the carbon chain from six to sixteen carbons, while KASII continues the elongation, converting 16:0-ACP to 18:0-ACP [39]. Each enzyme is specialized for specific chain length ranges during elongation. Our data indicate that *KASIII* was highly expressed during early seed development (10–17 DAF) and subsequently down-regulated (Figure 4 and Figure 5B; Table 2). The expression patterns of *KASI* and *KASII* were similar to that of *KASIII*, with high expression observed during early to mid-seed development (10–24 DAF), followed by down-regulation at later stages of seed development (31–38 DAF).

FATA and FATB are two different thioesterases with different functions: FATA primarily releases UFAs (e.g., C18:1) from ACP, whereas *FATB* terminates the biosynthesis of saturated or medium-chain FAs (e.g., C16:0) during lipid production [11,40]. In this study, we found that the expression of FATA was significantly higher (by approximately 10-fold) compared to *FATB* across all five developmental stages (Figure 5C), which likely promotes the accumulation of OA.

In addition, LACS catalyzes the formation of acyl-CoA from free FAs. A total of 10 *LACS* isoforms were identified, where *LACS7* (*Ug10240*) showed high expression at 31–38 DAF, *LACS9* (*Ug46285*) showed higher expression levels at the early stage (10–24 DAF), and *LACS7* (*Ug10240*) was up-regulated at the late stage (31–38 DAF) (Figure 5D, Table 2). The differential expression patterns of *LACS* suggest that the *LACS* gene family in sunflower may catalyze different substrates, regulate acyl-CoA synthesis, and facilitate the translocation of FAs from the plastid to the cytoplasm or ER.

#### 3.6.2. Genes Related to TAG and Oil Bodied in the Endoplasmic Reticulum

Diacylglycerol Acyltransferase (DGAT) catalyzes the final and key step in the Kennedy pathway of TAG synthesis. Based on their function and subcellular localization, DGAT enzymes are grouped into four subfamilies: DGAT1, DGAT2, DGAT3, and wax ester synthase (WS)/DGAT [41]. DGAT1 and DGAT2 are membrane-bound enzymes normally located in the ER, whereas DGAT3 is a soluble enzyme involved in a cytosolic pathway for TAG biosynthesis [42,43]. In this study, we identified three DGAT isoforms: DGAT1, DGAT2, and DGAT3. Among them, *DGAT2* (*Ug63054*) and *DGAT3* (*Ug21368*) showed higher expression levels at 38 DAF, while the other *DGAT2* (Ug69246) showed increased expression during the 10–24 DAF period (Table 2). Another enzyme, PDAT, catalyzes the acyl-CoA-independent synthesis of TAG. In Arabidopsis seeds, PDAT partially compensates for DGAT activity. Our results indicated that *PDAT2* (*Ug64137*) was expressed at significantly higher levels than *DGAT1*, *DGAT2*, and *DGAT3*, with peak expression observed at 10–24 DAF (Figure 5E), implying that PDAT activity may play a more critical role in the early stages.

In mature seeds, TAG is stored in specialized organelles called oil bodies. Oleosin (OLE), the predominant membrane protein in oil bodies, is essential for maintaining their shape and size by preventing fusion, thus ensuring their stability during seed development [44,45]. Caleosin (CLO), on the other hand, is involved in the formation and metabolism of oil bodies [46]. In this study, seven differentially expressed *OLE* genes were detected, five of which (*Ug67185*, *Ug63945*, *Ug29184*, *Ug31436*, and *Ug77732*) showed high expression levels during sunflower seed development (FPKM > 1000, with a maximum of 14,402.46). In addition, one *CLO*-encoding gene (*Ug391*) was consistently highly expressed throughout seed development (FPKM >1000 at 10 DAF, and FPKM >14,000 at 24 DAF) (Table 2).

#### 3.6.3. Genes Involved in Unsaturated Fatty Acid Synthesis

**Figure 6:**
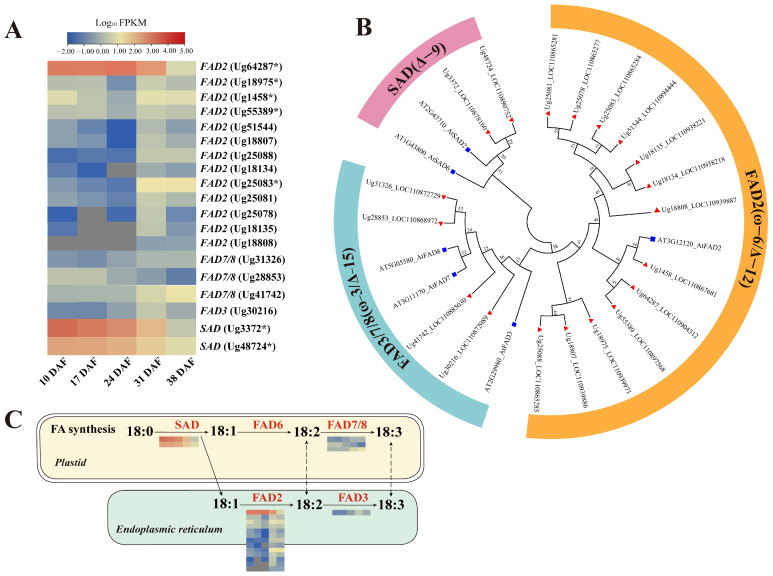
Fatty acid desaturase genes identified in developing sunflower seeds. (**A**) Heat map showing the expression levels of desaturase genes. (**B**) Phylogenetic analysis of desaturase genes in sunflower and Arabidopsis, performed using ClustalX2 for nucleotide sequence alignment and MEGA 7.0 Neighbor Joining (NJ) method for tree construction. (**C**) Pathway map illustrating the desaturase gene network. The asterisk (*) represents the major DEGs associated with FA synthesis.

The composition of mature sunflower embryos in this study shows that OA accounts for 54.72%, while LA accounts for 35.99%. The activity of FAD enzymes is critical in regulating OA levels. In addition, SAD is crucial for the de novo production of UFAs in plants [47]. Together, these enzymes modulate the proportion of UFAs in plant lipid biosynthesis. Transcriptomic analysis identified 19 *FAD* genes from sunflower RNA-seq data, including 13 *FAD2*, 2 *SAD*, 3 *FAD7/8*, and 1 *FAD3* (Figure 6A). Phylogenetic classification revealed distinct subfamily distributions: Δ9 desaturase (two members), ω-6/Δ12 desaturase (thirteen members), and ω-3/Δ15 desaturase (four members) (Figure 6B). Notably, two *SAD* genes (*Ug3372* and *Ug48724*) exhibited significant transcriptional activity between 10 and 24 DAF, with the *Ug3372* transcript showing particularly high expression levels (FPKM > 1000 at 10 DAF and FPKM > 500 at 17 DAF) (Table 2; Figure 5G). This period coincides with the accelerated phase of OA biosynthesis.

Thirteen *FAD2* unigenes were identified in the sunflower RNA-seq data, with the *FAD2-1* (*Ug64287*) showing significantly higher expression levels between 10 and 24 DAF (FPKM > 1000 at both 10 DAF and 24 DAF) compared to the other *FAD2* genes (Figure 5G; Table 2). This suggests that *FAD2-1* may play a key role in the biosynthesis of LA in sunflower. Previous studies have indicated that the low ALA content in sunflower seeds is closely related to the transcriptional regulation of Δ15-FAD during seed development [48]. In our study, the expression of *FAD3* (*Ug30216*) was significantly lower than that of *FAD2-1* during sunflower seed development (Figure 5E and Figure 6A,C). Since FAD3 encodes the Δ15-FAD responsible for the conversion of LA to ALA, the reduced expression of *FAD3* likely limits the conversion of LA, thereby contributing to the low ALA content observed in sunflower seeds. In addition, *FAD2-1* is involved in the conversion of OA to LA, and its higher expression compared to *FAD3* may further influence FA composition by increasing LA levels and limiting the accumulation of ALA.

### 3.7. Transcription Factors Related to Lipid Synthesis

**Figure 7:**
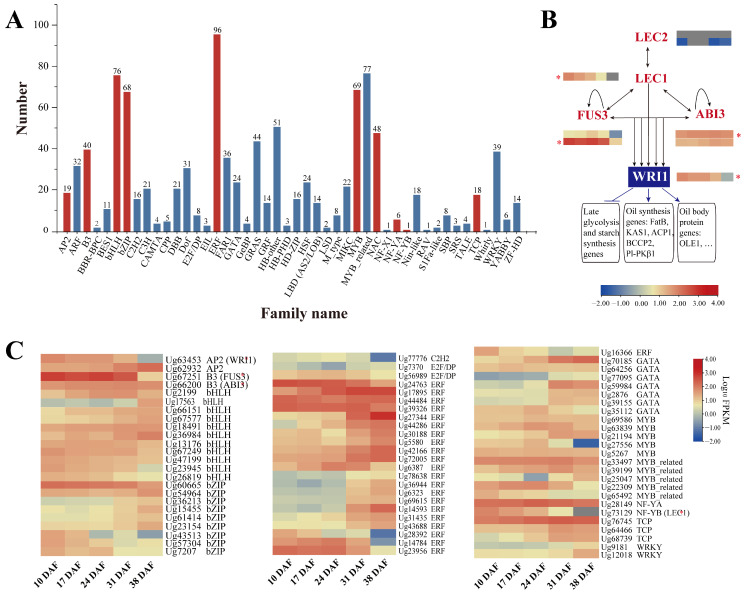
Transcription factors (TFs) involved in lipid synthesis in sunflower. (**A**) TFs identified in developing sunflower seeds. A total of 44 families were predicted, and the red columns indicate TFs that may be involved in FA and TAG biosynthesis. (**B**) Regulatory model of seed oil accumulation by TFs. (**C**) Heat map of differential expression of TF genes with FA and TAG biosynthesis. The asterisk (*) represents the major DEGs associated with TAG synthesis.

TFs are crucial for regulating multiple biological processes throughout plant growth and development. In this study, 1031 DEGs belonging to 46 different TF families were identified as TFs. Among them, nineteen *AP2*, forty *B3*, seventy-six *bHLH*, sixty-eight *bZIP*, ninety-six *ERF*, sixty-nine *MYB*, forty-eight *NAC*, six *NF-YA*, one *NF-YB*, and eighteen *TCP* members were potentially associated with lipid biosynthesis (Figure 7A; Table S7). Among them, eight TFs were identified as key regulators of seed development and oil accumulation, including *LEC1*, *LEC2*, *FUS3*, *ABI3*, and *WRI1*. The transcriptional regulatory network of oil biosynthesis in sunflower seeds is shown in Figure 7B. The *LEC2* gene, a B3 domain TF essential for embryo development [49], was identified, but its expression (*Ug68124* and *Ug68126*) was nearly undetectable throughout sunflower seed development (Table 3; Figure 7C), suggesting that *LEC2* may have a minimal effect on seed oil accumulation.

The expression of *LEC1* (*Ug73129*), an NF-YB family member that regulates FA biosynthesis, peaked during early to mid-seed development (10–24 DAF) and declined at later stages (31–38 DAF), mirroring the trend of FA accumulation. Similarly, the AP2 TF *WRI1* (*Ug63453*), a key regulator of glycolysis and FA metabolism, was up-regulated during 10–24 DAF, coinciding with rapid seed oil accumulation. The B3 TFs *ABI3* and *FUS3* were also identified, with *FUS3* (*Ug67251*) showing significantly higher expression during 10–31 DAF. Notably, *FUS3* exhibited much higher expression than *LEC1*, *LEC2*, *WRI1*, and *ABI3* (Figure 5H), suggesting its critical role in sunflower seed development and TAG synthesis.

In addition to the well-known TFs regulating TAG biosynthesis, several other TFs, including *MYBs*, *ERFs*, *bZIPs*, *bHLHs*, *WRKYs*, and *TCPs*, showed high expression during sunflower seed development (Figure 7C). This suggests their potential involvement in FA biosynthesis.

### 3.8. qPCR Analysis of DEGs Involved in Lipid Metabolism

**Figure 8:**
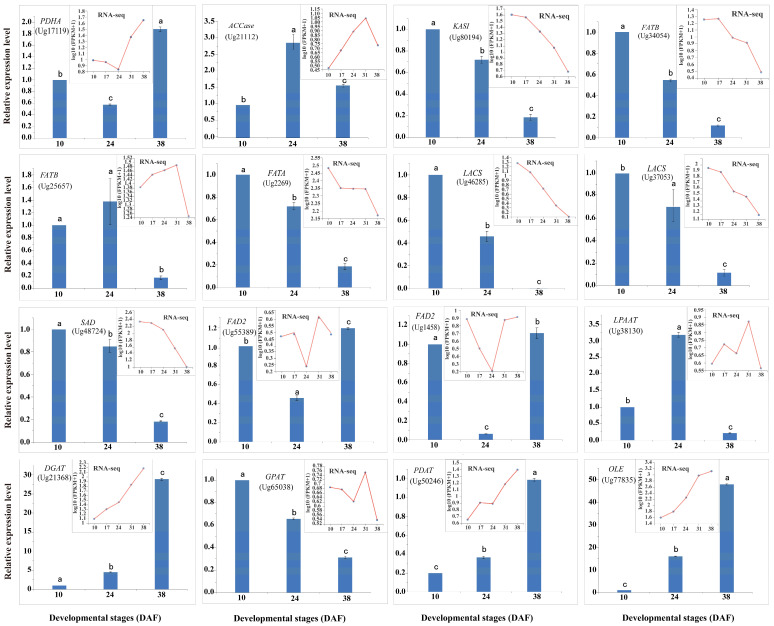
Expression validation of genes between RT-qPCR and RNA-seq at three stages of seed development in sunflower. Red line and blue volume represent RNA-seq and data, respectively. Sunflower 18S rRNA gene (AF1057577) was used as an internal control. Means with different letters indicate a significant difference (*p* < 0.05); error bars lines indicate SD (*n* = 3).

To confirm the expression patterns at different developmental stages revealed by the transcriptomic analysis, RT-qPCR was performed on 16 DEGs involved in lipid metabolic pathways. These genes included those involved in FA biosynthesis (PDHA, ACC, KASI, FATA, FATB, SAD, LACS, FAD2), TAG assembly (GPAT, LPAAT, DGAT, PDAT), and lipid storage regulation (OLE). RNA samples were collected from sunflower seeds at three critical developmental stages: early embryogenesis (10 DAF), mid-maturation (24 DAF), and late maturation (38 DAF). The RT-qPCR results for these genes closely matched the RNA-seq data (Figure 8), confirming the accuracy of the transcriptome analysis.

## 4. Discussion

### 4.1. Role of Key De Novo Biosynthetic Enzymes in the Regulation of Fatty Acid Composition in Sunflower Seeds

During early seed development (10–17 DAF), several genes involved in de novo FA biosynthesis were found to have significantly up-regulated expression, including the plastidial FAS complex—*ACC*, *ENR*, *MCAT*, *HAD*, *FATA*, *KASI*, *KASⅡ*, and *KAS III* (Table 2). The ACC enzyme initiates this process by catalyzing the ATP-dependent conversion of acetyl-CoA to malonyl-CoA, a rate-limiting step regulated by biotinylation [50]. The enzymes KASI, KASII, and KASIII are involved in acyl chain elongation, whereas HAD stabilizes acyl-ACP intermediates to ensure efficient FA biosynthesis. Overexpression of *JcKASII* from *Jatropha curcas* in Arabidopsis caused a decline in 16-carbon FAs and a rise in 18-carbon FAs in both leaves and seeds [51]. Similarly, overexpression of *JcKASIII* in Arabidopsis resulted in higher PA content and an increased proportion of C16-C18 FAs in seeds [52]. Overall, these findings indicate that both *JcKASII* and *JcKASIII* play critical roles in regulating FA composition, with *JcKASII* primarily affecting the balance between C16 and C18 FAs, whereas *JcKASIII* promotes the accumulation of PA in seeds. Our data showed that the expression of *KASI*, *KASII*, and *KASIII* was significantly higher during the early stages of sunflower seed development, suggesting their important roles in the early accumulation of FAs in sunflower seeds. However, their functions and mechanisms require further investigation.

FATA encodes fatty acyl-ACP thioesterase A, which primarily catalyzes the substrate C18:1-ACP, whereas FATB has a higher catalytic activity towards saturated acyl-ACPs [11,37]. In Arabidopsis *fata2* mutants, except for a slight decrease in C24:0 and no change in C22:0 levels, all other FAs increased by 10% to 60% [53]. In Arabidopsis double mutants in which both *FATA* gene copies were knocked out, the TAG content of the seeds was reduced, while the percentage of ALA and erucic acid in the total FA composition increased [54]. In this study, the sunflower *FATA* gene was highly expressed throughout seed development (Figure 5C), while the *FATB* gene showed lower expression levels. This suggests that sunflower *FATA* may play a crucial role in de novo FA biosynthesis, contributing to the increased production of OA.

### 4.2. Role of FAD and SAD Enzymes in Regulating Sunflower Seed Fatty Acid Desaturation and Composition

In plant lipid metabolism, the synthesis of PUFAs mainly occurs in the plastids and the ER. In plastids, two desaturase systems catalyze sequential reactions: FAD6 introduces a double bond at the Δ12 position of OA, converting it to LA, while FAD7/FAD8 further desaturates LA at the Δ15 position to produce α-ALA [18]. Both of these reactions use ferredoxin as the primary electron donor [55]. In the ER, both FAD2 and FAD3 catalyze the desaturation of OA to LA, and then further to ALA, with cytochrome b5 as the primary electron donor [56]. Regulation of FAD activity significantly affects the accumulation of OA, LA, and ALA in the seeds of several plant species, such as *Camelina sativa* [57], *Glycine max* [58], *Paeonia section* [59], *Zanthoxylum bungeanum* [60], *Linum usitatissimum* [61], and *Eucommia ulmoides* [62]. In this study, 13 *FAD2* genes were determined to be active in the transcriptome of the sunflower seed, with *FAD2-1* (*Ug64287*) returning significantly higher levels of expression than the other identified *FAD2* genes. This suggests that *FAD2-1* is the major contributor to FAD2 enzyme activity in sunflower seeds to highlight its critical role in converting OA to LA. In addition, the expression levels of sunflower *FAD3* and *FAD7*/*FAD8* were extremely low during seed development and FA accumulation, which probably explains the minimal content of ALA in mature sunflower seeds.

SAD enzymes are responsible for the initial desaturation step, converting SFAs to the MUFA (C18:1), which can be further converted into PUFAs. Therefore, SADs are crucial for the regulation of seed oil content and composition. In Arabidopsis, seven candidate *SAD* genes were identified, four of which (*AAD1*, *AAD5*, *AAD6*, and *FAB2*) showed expression patterns related to the accumulation of OA and its derivatives during seed development [63]. In this study, two *SAD* genes were expressed in developing sunflower seeds, with one gene (*Ug3372*) showing particularly high expression between 10 and 24 DAF (Table 2; Figure 5G), suggesting its key role in UFA biosynthesis. Given the established role of *SAD* genes in converting SFAs to MUFAs [48,64,65], it is reasonable to propose that the increased expression of *SAD* genes could enhance enzymatic activity, thereby facilitating a greater accumulation of MUFAs.

### 4.3. Key Enzymes and Transcription Factors Regulating Lipid Accumulation in Sunflower Seed

In the transcriptomic data of developing sunflower seeds, we identified key genetic factors that regulate TAG biosynthesis, shedding light on stage-specific regulatory mechanisms that control lipid accumulation. In particular, genes such as *LACS7*, *LPCAT*, *DGAT2*, and *PDAT2* appear to play critical roles in TAG synthesis. LACS catalyzes the activation of FAs to form acyl-CoA, a key precursor in TAG synthesis. LPCAT facilitates the acylation of lysophosphatidylcholine to phosphatidylcholine, a critical step in the formation of DAG. DGAT is recognized as a critical enzyme in the conversion of DAG to TAG; therefore, it is thought to be the rate-limiting enzyme in the accumulation of storage lipids in plants [66]. Several studies have shown that DGAT is critical in regulating both the amount and type of FAs incorporated into storage TAGs [67,68,69]. As a result, genes encoding DGAT-like proteins have become key targets in biotechnological strategies aimed at improving oil content and FA profiles in oilseed crops.

The DGAT enzyme family consists of three major isoforms (DGAT1, DGAT2, DGAT3) with different functions in plant lipid metabolism. In most plants, DGAT1 is more extensively involved in lipid synthesis, whereas DGAT2 primarily regulates the accumulation of specialized FAs. However, their functions are not mutually exclusive [70]. DGAT2 acts upstream of the DGAT1 and influences the synthesis and storage of TAG [71]. Heterologous expression of *Neurospora crassa DGAT2* with a shorter form (*S-NcDGAT2*) in maize increased oil content, altered FA composition, increased OA content, and decreased LA content [72]. In our study, *DGAT1* expression remained low throughout the development of sunflower seeds. In contrast, *DGAT2* (*Ug69246*) showed higher expression during the early and middle stages (10–24 DAF), while *DGAT2* (*Ug63054*) was more highly expressed during the later stages (31–38 DAF) (Table 2). These expression patterns suggest that *DGAT2* may play a more critical role in oil accumulation than *DGAT1*. However, further studies are needed to fully understand the functional differences between these two genes.

The PDAT enzyme in plants catalyzes the transfer of acyl groups from phospholipids to DAG, providing an alternative pathway for TAG biosynthesis. This pathway complements the conventional DGAT-mediated pathway and plays a critical role in lipid remodeling and storage, especially under stress conditions or during seed development [73,74,75]. DGAT1 and PDAT1 have overlapping functions in TAG biosynthesis in Arabidopsis, and both enzymes are crucial for the proper development of pollen and seeds [76]. However, PDAT might have a more significant function in other plant species. In seeds that produce highly unsaturated oils, such as sunflower, safflower [77], flax [78], and cotton [79], the capacity of PDAT to use phosphatidylcholine, a substrate involved in desaturation, as a direct precursor for TAG synthesis is particularly beneficial. In this study, sunflower *PDAT2* exhibited significantly higher expression than the *DGAT* isoforms, with the highest expression observed during early and mid-stages (10–24 DAF), which corresponds to the period of rapid TAG accumulation in seeds. This suggests that the *PDAT*-mediated pathway of TAG synthesis plays a critical role in lipid accumulation in sunflower seeds.

In addition, several regulatory factors, including *WRI1*, *LEC1*, *ABI3*, and *FUS3*, were identified as up-regulated during the early and mid-stages (10–24 DAF), which coincides with the rapid accumulation of oil in sunflower seeds, suggesting their important roles in regulating lipid biosynthesis and storage. WRI1 is known to activate genes involved in FA and TAG biosynthesis, while LEC1, ABI3, and FUS3 are key regulators of seed development and lipid accumulation, controlling genes responsible for lipid metabolism [28,80,81,82]. These TFs coordinate the pathways that drive the accumulation of storage lipids in seeds. In addition, other TF families, including *MYBs*, *ERFs*, *bZIPs*, *bHLHs*, *WRKYs*, and *TCPs*, also showed higher expression during sunflower seed development. These candidate TFs may play important roles in sunflower lipid metabolism and provide novel regulatory mechanisms for oil accumulation. The identification of these TFs provides new targets for improving oil yield and quality in sunflower through biotechnology and breeding strategies.

This study provides valuable insights into the molecular mechanisms of fatty acid synthesis and triacylglycerol (TAG) accumulation in sunflower seeds, with the aim of improving oil yield and quality. Key genes such as FAD2-1, SAD, and FATA play important roles in the regulation of fatty acid composition and contribute to the high content of unsaturated FAs in ‘T302’ oil, especially LA and OA. The discovery of key transcription factor genes such as WRI1, LEC1, and ABI3, which regulate TAG synthesis during seed maturation, provides new targets for genetic modification. These findings have improved our understanding of lipid metabolism and provide potential genetic targets for improving oil content and modifying FA profiles in sunflower and other oilseed crop breeding.

## 5. Conclusions

In conclusion, this study provides valuable insights into the molecular mechanisms of lipid biosynthesis and oil accumulation in oilseed sunflower seeds. We have identified key genes and transcription factors involved in fatty acid synthesis, triacylglycerol formation, and lipid storage, highlighting potential targets for improving oil yield and quality. Future research should focus on the functional roles of these genes and transcription factors and explore their potential for optimizing lipid accumulation under different environmental conditions. While this study presents significant findings, it is important to acknowledge that we used only the five developmental stages of sunflower seeds, and environmental factors may influence the results, and further studies are needed to validate these findings under different conditions and larger sample sets.

## Figures and Tables

**Table 1:** Fatty acid composition (% of total fatty acids) in mature seeds of ‘T302’ sunflower cultivar.

Fatty Acids	Formula	Percentage (%)
lauric acid	C12:0	0.01
myristic acid	C14:0	0.07
pentadecanoic acid	C15:0	0.02
palmitic acid	C16:0	7.46
heptadecanoic acid	C17:0	0.06
stearic acid	C18:0	5.04
arachidonic acid	C20:0	0.36
eicosanoic acid	C21:0	0.01
behenic acid	C22:0	0.03
pantotrizoic acid	C23:0	0.04
lignoceric acid	C24:0	0.29
myristic acrylic acid	C14:1N5	0.07
pentadecenoic acid	C15:1N5	0.06
palmitoleic acid	C16:1N7	0.13
heptadecenoic acid	C17:1N7	0.04
trans-oleic acid	C18:1TN9	0.03
oleic acid	C18:1N9	37.25
linoleic acid	C18:2N6	48.47
linolenic acid	C18:3N3	0.06
eicosenoic acid	C20:1N9	0.15
eicosapentaenoic acid	C20:5N3	0.34
docosapentaenoic acid	C22:5N6	0.01
tetracosenoic acid	C24:1N9	0.01
docosahexaenoic acid (DHA)	C22:6N3	0.01
Total_ SFAs		13.39
Total_ MUFAs		37.73
Total_ PUFAs		48.88

Note: The first double bonds in unsaturated fatty acids, designated n3, n5, n6, n7, and n9, are located at positions 3, 5, 6, 7, and 9, respectively, from the methyl end of the carbon chain. SFAs, saturated fatty acids; MUFAs, monounsaturated fatty acids; PUFAs, polyunsaturated fatty acids.

**Table 2:** Identification of lipid-related genes in the developing sunflower embryo.

Enzyme	KEGG Annotation	Ug ID	Gene Name	10 DAF	17 DAF	24 DAF	31 DAF	38 DAF	Model
ACC/α-CT	acetyl-coenzyme A carboxylase carboxyl transferase subunit α	*Ug40664*	LOC110884458	52.17	38.65	22.77	19.89	13.02	blue
		*Ug65667*	LOC110909894	**103.79**	62.47	33.27	15.24	2.2	blue
ACC/BC	biotin carboxylase 2, chloroplastic-like	*Ug80390*	LOC110920936	96.35	44.51	25.83	11.01	2.84	blue
		*Ug4062*	LOC110882513	**149.27**	76.77	49.89	28.14	11.67	blue
ACC/BCCP	biotin carboxyl carrier protein of acetyl-CoA carboxylase, chloroplastic-like	*Ug5997*	LOC110917264	25.373	15.25	7.36	6.19	3.49	blue
		*Ug38366*	LOC110879644	**237.86**	**212.6**	**132.74**	60.93	9.06	blue
		*Ug71124*	LOC110915869	62.46	38.75	20.41	11.74	4.26	blue
MCAT	malonyl CoA-acyl carrier protein transacylase-like	*Ug1819*	LOC110869205	**110.83**	**106.79**	72.17	53.85	14.11	blue
HAD	3-hydroxyacyl-[acyl-carrier-protein] dehydratase	*Ug9482*	LOC110928978	**162.82**	96.32	37.41	8.29	0.94	blue
ENR/FABI	enoyl-[acyl-carrier-protein] reductase [NADH]	*Ug8057*	LOC110927155	11.85	9.85	10.04	52.21	43.95	turquosio
		*Ug80148*	LOC110920857	**103.98**	48.75	33.04	10.11	1.25	blue
		*Ug74540*	LOC110918310	77.22	39.44	21.94	11.02	3.84	blue
KAR	3-oxoacyl-ACP reductase	*Ug76007*	LOC110926295	94.51	57.81	44.01	29.67	22.61	blue
		*Ug45295*	LOC110886898	74.61	41.17	23.86	15.71	10.65	blue
KASI	3-oxoacyl-[acyl-carrier-protein] synthase I	*Ug80194*	LOC110925958	**231.82**	**137.51**	84.1	55.46	14.97	blue
		*Ug16903*	LOC110937383	39.87	35.72	20.45	12.02	3.97	blue
		*Ug75867*	LOC110923177	1.1	1.39	0.99	1.84	0.73	brown
KASII	3-oxoacyl-[acyl-carrier-protein] synthase II	*Ug33622*	LOC110877021	**191.49**	177	**105.5**	50.4	3.36	blue
		*Ug49585*	LOC110891189	10.87	7.85	4.83	4.87	6.25	yellow
KASIII	3-oxoacyl-[acyl-carrier-protein] synthase III	*Ug21332*	LOC110941199	**124.88**	75.5	48.56	32.63	5.19	blue
		*Ug7042*	LOC110913024	15.96	11.36	5.54	3.92	0.96	blue
FAD2	delta (12) fatty acid desaturase	*Ug64287*	LOC110904312	**1022.51**	**994.4**	**1304.58**	**346.14**	5.73	blue
		*Ug25083*	LOC110865284	0.26	0.1	0.05	18.88	20.01	turquosio
		*Ug1458*	LOC110867681	7.07	2.2	0.65	8.93	7.59	turquosio
		*Ug55389*	LOC110897568	1.96	2.17	0.75	3.08	2.2	turquosio
		*Ug18975*	LOC110939971	1.72	1.85	0.16	3.43	1.66	grey
SAD	stearoyl-[acyl-carrier-protein] 9-desaturase	*Ug3372*	LOC110878160	**1693.36**	**934.07**	**470.82**	84.21	3.08	blue
		*Ug48724*	LOC110890752	**192.41**	**174.93**	**113.85**	40.04	9.64	blue
FATA	oleoyl-acyl carrier protein thioesterase	*Ug2269*	LOC110871748	**306.52**	**225.67**	**221.38**	224.42	156.45	turquosio
FATB	palmitoyl-acyl carrier protein thioesterase	*Ug34054*	LOC110877217	16.91	17.45	8.78	7.46	2.19	blue
LACS	long chain acyl-CoA synthetase 1	*Ug65810*	LOC110910053	2.13	3.87	6.94	24.48	18.81	turquosio
	long chain acyl-CoA synthetase 2	*Ug43869*	LOC110886089	16.23	27.99	30.52	10.7	1.57	blue
	long chain acyl-CoA synthetase 4	*Ug11892*	LOC110930246	36.09	38.02	25.77	23.43	8.24	blue
		*Ug58965*	LOC110899500	0	0.51	0.39	3.69	5.33	turquosio
	long chain acyl-CoA synthetase 6, peroxisomal	*Ug31054*	LOC110872567	0.02	0.07	0	0.12	40.11	turquosio
		*Ug42142*	LOC110885243	7.33	5.63	2.58	3.86	2.65	yellow
	long chain acyl-CoA synthetase 7, peroxisomal	*Ug10240*	LOC110929342	17.95	29.45	26.21	**139.35**	**140.43**	turquosio
		*Ug1412*	LOC110895747	2.15	1.38	2.51	4.92	0.47	brown
	long chain acyl-CoA synthetase 9, chloroplastic-	*Ug37053*	LOC110878808	18.53	10.99	4.26	1.53	0.28	blue
		*Ug46285*	LOC110889498	84.03	72.58	34.41	28.61	13.77	blue
GPAT	glycerol-3-phosphate acyltransferase	*Ug65669*	LOC110909897	3.95	5.02	8.88	9.99	1.14	brown
		*Ug64602*	LOC110904530	1.67	0.84	0.84	2.89	0.69	brown
		*Ug65083*	LOC110904838	3.92	3.76	3.24	4.68	2.62	brown
		*Ug60504*	LOC110900391	4.92	6.71	9.99	12.04	7.39	brown
		*Ug24261*	LOC110864776	5.86	10.34	10.34	13.55	7.49	green
ATS1	glycerol-3-phosphate acyltransferase, chloroplastic	*Ug11854*	LOC110930224	1.29	0.96	0.83	4.61	4.85	turquosio
		*Ug59423*	LOC110899775	6.57	5.32	4.42	5	8.2	grey
		*Ug70832*	LOC110916084	1.38	2.01	0.63	1.57	7.94	turquosio
LPAAT	lysophosphatidic acid acyltransferase	*Ug55427*	LOC110897582	26.12	13.03	12.27	7.04	1.9	blue
		*Ug38130*	LOC110879498	2.97	4.31	3.61	6.47	2.69	green
		*Ug43812*	LOC110886059	2.41	1.74	1.55	1.92	1.55	blue
		*Ug69414*	LOC110912534	10.01	8.46	4.57	16.5	33.87	turquosio
		*Ug63749*	LOC110903942	2.68	3.53	3.67	10.19	16.89	turquosio
PAP	phosphatidate phosphatase PAH1-like	*Ug3797*	LOC110880605	4.51	4.41	3.01	3.11	1.43	blue
		*Ug49621*	LOC110891213	1.38	1.87	2.28	5.22	7.4	turquosio
		*Ug38050*	LOC110879450	2.91	4.93	5.27	12.33	9.89	turquosio
		*Ug5507*	LOC110914598	2.62	3.99	5.01	7.52	4.91	turquosio
		*Ug62420*	LOC110908856	2.38	3.21	3.5	9.06	15.91	turquosio
PDAT	phospholipid: diacylglycerol acyltransferase 1	*Ug44521*	LOC110886458	1.22	1.45	1.31	8.07	15.62	turquosio
		*Ug50246*	LOC110893689	3.61	7.15	6.85	14.4	24.3	turquosio
	phospholipid: diacylglycerol acyltransferase 2	*Ug64137*	LOC110904203	**319.61**	**314.75**	**321.83**	**176.02**	46.18	blue
LPCAT	lysophospholipid acyltransferase	*Ug32561*	LOC110873391	1.35	3.28	1.93	10.18	12	turquosio
		*Ug6933*	LOC110922258	70.16	43.99	25.82	14.16	7.09	blue
		*Ug5650*	LOC110915451	14.74	16.7	18.71	40.04	72.81	turquosio
DGAT	diacylglycerol O-acyltransferase 1	*Ug45411*	LOC110886978	0.13	0.46	0.49	4.05	3.91	turquosio
		*Ug50291*	LOC110893727	5.5	7.51	4.45	8.21	4.79	yellow
	diacylglycerol O-acyltransferase 2	*Ug69246*	LOC110912448	37.46	30.43	41.78	27.58	5.05	blue
		*Ug63054*	LOC110909196	3.39	9.05	23.09	84.76	**125.24**	turquosio
	diacylglycerol O-acyltransferase 3, cytosolic	*Ug21368*	LOC110941225	12.08	19.43	27.61	69.91	**155.47**	turquosio
CPI	cycloeucalenol cycloisomerase	*Ug38774*	LOC110879898	8.71	7.21	6.3	6.9	**3.62**	blue
CPT	choline/ethanolaminephosphotransferase	*Ug45630*	LOC110889136	18.58	16.5	14.76	27.11	9.44	brown
		*Ug5123*	LOC110912689	1.75	2.54	3.01	7.18	2.73	turquosio
PLD	phospholipase D	*Ug13661*	LOC110935648	22.04	28.03	19.28	69.34	**119.35**	turquosio
		*Ug43122*	LOC110885693	1.6	1.4	0.37	1.3	1.167	yellow
		*Ug36550*	LOC110878503	23.17	28.45	22.51	56.19	**113.88**	yellow
		*Ug54911*	LOC110897355	4.35	2.84	1.22	2.12	3.1	yellow
		*Ug50834*	LOC110894046	2.89	4.22	3.61	6.29	1.32	brown
		*Ug41664*	LOC110884979	0.7	1.19	1.73	2.55	2.77	turquosio
		*Ug52043*	LOC110894725	0.37	0.76	0.75	3.65	2.12	turquosio
		*Ug60454*	LOC110900357	1.18	2.25	1.69	2.66	0.88	brown
Caleosin (CLO)	peroxygenase	*Ug391*	LOC110943549	**1628.64**	**2634.68**	**4621.25**	**4012.81**	**1703.27**	turquosio
		*Ug80554*	LOC110922725	1.45	1.3	2.37	7.53	2.97	turquosio
		*Ug80552*	LOC110922319	12.52	11.84	27.84	44.47	12.47	green
oleosin (OLE)	oleosin	*Ug67185*	LOC110911264	**2918.2**	**4725.85**	**10,221.61**	**11,148.61**	**3956.6**	green
		*Ug65317*	LOC110906283	**681.18**	**686.94**	**1068.37**	**480.97**	**340.3**	green
		*Ug63945*	LOC110904071	**7917.12**	**7774.56**	**9851.5**	**7118.1**	**3225.92**	green
		*Ug29184*	LOC110869166	**10,678.39**	**14,402.46**	**21,354.37**	**10,587.85**	**1985.85**	green
		*Ug31436*	LOC110871077	**1858.28**	**1716.85**	**2878.29**	**1815.63**	**2101.36**	green
		*Ug71463*	LOC110919066	**241.72**	**503.79**	**1575.6**	**610.37**	**429.39**	green
		*Ug77732*	LOC110921783	**4105.23**	**5143.99**	**7891.14**	**3065.98**	**1777.43**	green

Note: Unigenes exhibiting FPKM values exceeding 100 are emphasized using bold formatting.

**Table 3:** Expression profiling of lipid metabolism-regulating transcription factors during sunflower seed maturation.

Enzyme	KEGG Annotation	Ug ID	Gene Name	10 DAF	17 DAF	24 DAF	31 DAF	38 DAF
LEC1	nuclear transcription factor Y subunit B-6-like (NF-YB)	Ug73129	LOC110918598	78.70	39.24	20.43	4.24	0
LEC2	B3 domain-containing transcription factor LEC2-like	Ug68124	LOC110909750	0.02	0	0	0.02	0.04
		Ug68126	LOC110913834	0	0	0	0	0
FUS3	B3 domain-containing transcription factor FUS3-like	Ug64049	LOC110906026	4.23	5.43	10.28	5.05	0.16
		Ug67251	LOC110911305	**630.63**	**419.49**	**573.11**	**297.36**	8.39
ABI3	B3 domain-containing transcription factor ABI3-like	Ug66200	LOC110910470	45.38	60.76	59.76	66.66	57.29
		Ug39718	LOC110883938	19.18	17.90	24.46	35.48	30.41
WRI1	ethylene-responsive transcription factor WRI1	Ug63453	LOC110903740	65.87	41.94	42.60	19.73	0.58

Note: Unigenes exhibiting FPKM values exceeding 100 are emphasized using bold formatting.

## Data Availability

All the data in this study are included in this manuscript. The raw RNA-seq reads have been deposited in the NCBI database (accession PRJNA1220589).

## Supplementary Materials

The following supporting information can be downloaded at: https://www.mdpi.com/article/10.3390/genes16040393/s1, Supplementary figures: Figure S1: Size distribution of sunflower Illumina reads. Figure S2: Function annotation statistics table. Figure S3: COG classification of the identified unigenes. Figure S4: GO functional analysis of the DEGs. Figure S5: Bubble charts of GO (A) and KEGG (B) analyses. Table S1: Primers used in this study. Table S2: Annotation information of 81676 genes. Table S3: Expression of 23,396 differentially expressed genes per period and differential expression. Table S4: Enrichment pathways and number of genes associated with lipid metabolism classification in sunflower seeds. Table S5: KEGG enrichment analysis of each module in WGCNA. Table S6: Enzymes/proteins related to lipid accumulation in sunflower seeds. Table S7: Transcription factors associated with lipid accumulation in sunflower seeds.
