# Supplementary material for: Transcriptome Analysis Reveals Key Genes Involved in Fatty Acid and Triacylglycerol Accumulation in Developing Sunflower Seeds
Source: Genes (Basel). 2025 Mar 29;16(4):393. doi: 10.3390/genes16040393 (PMC12026707; doi:10.3390/genes16040393)
Supplement: Supplementary file 1 [file genes-16-00393-s001.zip › Supplementary figures.pdf]

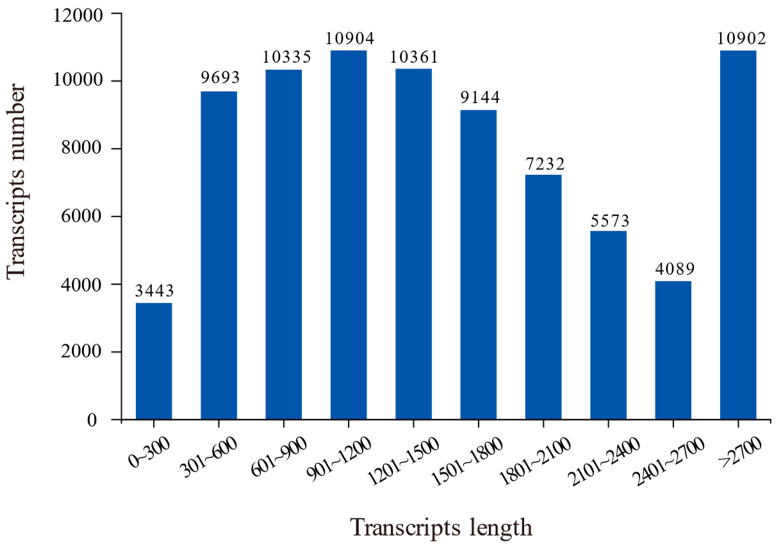

Figure S1 Size distribution of sunflower Illumina reads. The x-axis corresponds to the distribution of sequence lengths in base pairs (bp), while the y-axis indicates the frequency of sequences within each specific length interval.

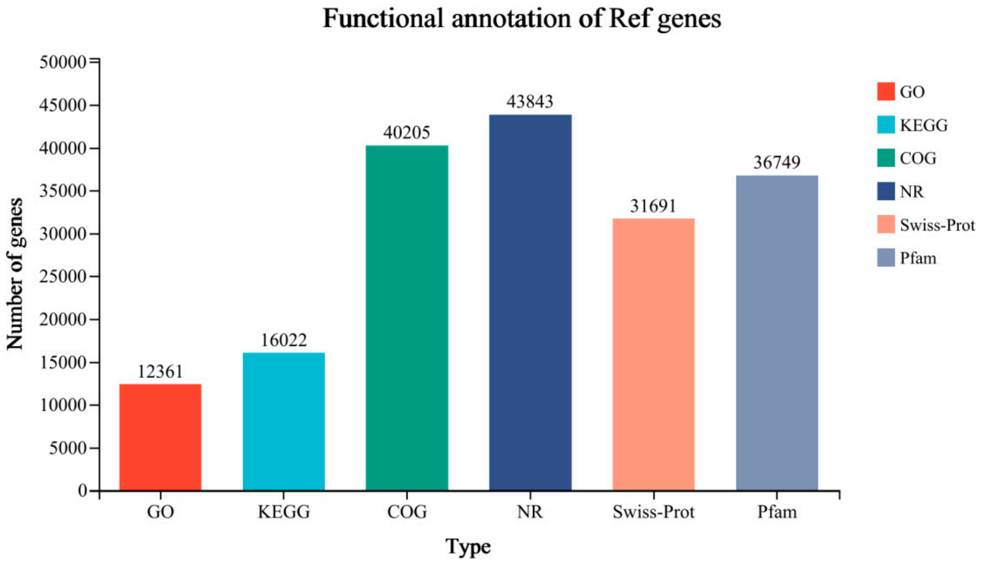

Figure S2 Function annotation statistics table. The x-axis denotes the database name, while the y-axis represents the number of genes annotated to the respective database.

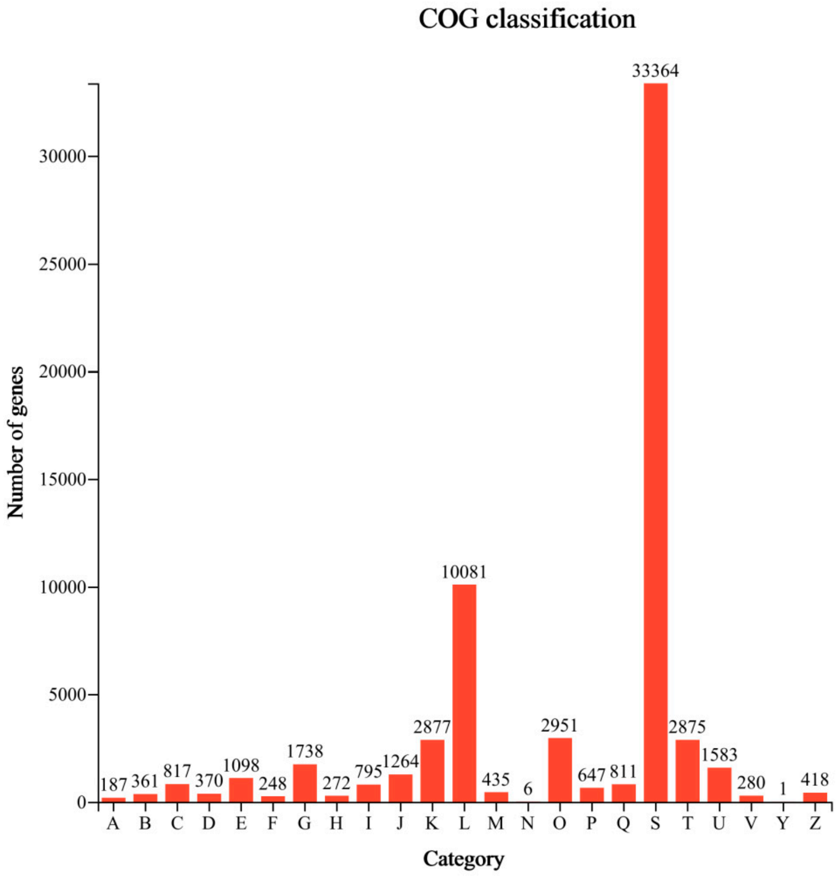

Figure S3 COG classification of the identified unigenes. A. RNA processing and modification; B. Chromatin structure and dynamics; C. Energy production and conversion; D. Cell cycle control, cell division, chromosome partitioning; E. Amino acid transport and metabolism; F. Nucleotide transport and metabolism; G. Carbohydrate transport and metabolism; H. Coenzyme transport and metabolism; I. Lipid transport and metabolism; J. Translation, ribosomal structure and biogenesis; K. Transcription; L. Replication, recombination and repair; M. Cell wall/membrane/envelope biogenesis; N. Cell motility; O. Posttranslational modification, protein turnover, chaperones; P. Inorganic ion transport and metabolism; Q. Secondary metabolites biosynthesis, transport and catabolism; S. Function unknown; T. Signal transduction mechanism; U. Intracellular trafficking, secretion, and vesicular transport; V. Defense mechanisms; Y. Nuclear structure; Z. Cytoskeleton.

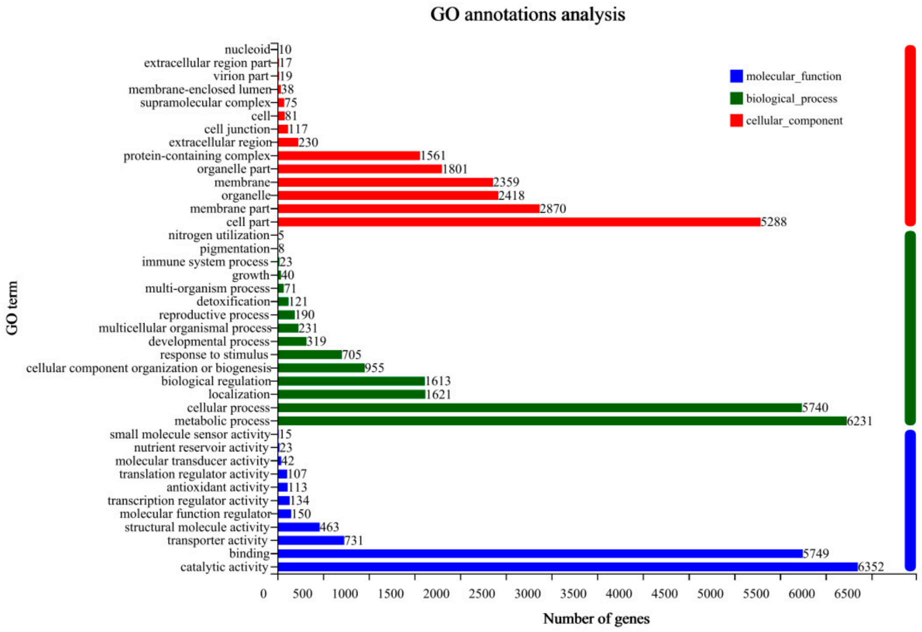

Figure S4 GO functional analysis of the DEGs.

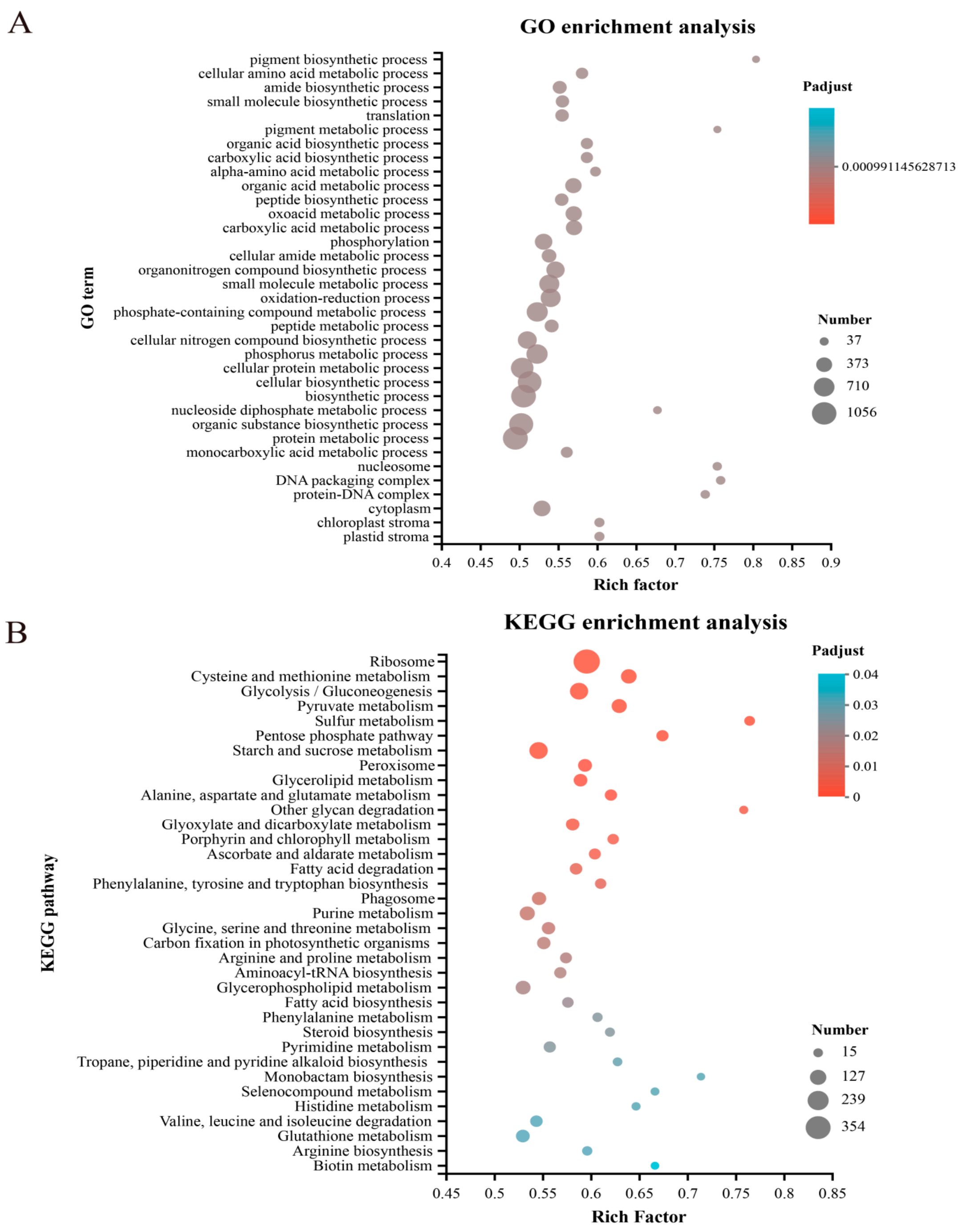

Figure S5 Bubble charts of GO (A) and KEGG (B) analyses. The y-axis represents the pathway names. The x-axis corresponds to the Rich factor. The dot size reflects the gene count within each pathway, and the dot color signifies the range of adjusted p-values (padjust). Enrichment results are visualized by default at padjust <0.5).
